# “It’s about time they taught us”: a qualitative study evaluating the barriers to finding and screening child contacts under five at risk for TB in Gauteng Province, South Africa from the provider and caregiver perspectives

**DOI:** 10.1186/s12913-023-10359-0

**Published:** 2023-12-15

**Authors:** Fadzai Munedzimwe, C. M. Chetty-Makkan, R. Mukora, S. Charalambous, K. Turner, V. Chihota

**Affiliations:** 1 The Aurum Institute, Aurum House, The Ridge, 29 Queens Road, Parktown, Johannesburg, 2193 South Africa; 2 School of Public Health, University of Witwatersrand, Johannesburg, South Africa; 3 Health Economics and Epidemiology Research Office, Wits Health Consortium, Faculty of Health Sciences, University of the Witwatersrand, Johannesburg, South Africa; 4 Yale University, New Haven, Connecticut, USA; 5 Division of Infectious Diseases, Department of Medicine, Vanderbilt University School of Medicine, Nashville, TN USA

**Keywords:** Childhood tuberculosis, Child household contact, Screening, TB preventive therapy, Qualitative

## Abstract

**Background:**

Inadequate numbers of children under five years of age who are exposed to tuberculosis (TB) in the home (child contact) are initiated on TB preventive treatment (TPT) in South Africa. We assessed barriers of initiating isoniazid preventive therapy (IPT) in this age group.

**Methods:**

We conducted a qualitative study at two primary health clinics in the Ekurhuleni district in Gauteng Province. Between April and July 2019, we enrolled facility managers, TB staff and parents or legal guardians of child contacts (caregivers) attending for care, at the two facilities. Semi-structured questionnaires, facility observations and in-depth interviews using a semi-structured interview guide were used to collect data. Findings from the semi-structured questionnaires with facility staff and facility observations were summarized. Thematic analysis with a deductive approach was used to analyse the data from the in-depth interviews with caregivers.

**Results:**

Two facility managers took part in the study and were assisted to complete the semi-structured questionnaires by TB staff. Fifteen caregivers aged between 18 and 43 years were interviewed of which 13 (87%) were female. Facility managers and TB staff (facility staff) felt that even though caregivers knew of family members who were on TB treatment, they delayed bringing their children for TB screening and TPT. Facility staff perceived caregivers as not understanding the purpose and benefits of TB prevention strategies such as TPT. Caregivers expressed the desire for their children to be screened for TB. However, caregivers lacked knowledge on TB transmission and the value of TB prevention in children at high risk of infection.

**Conclusion:**

While facility staff perceived caregivers to lack responsibility, caregivers expressed limited knowledge on the value of screening their children for TB as reasons for not accessing TB preventive services. Health education on TB transmission, screening, and TB prevention strategies at a community level, clinics, creches, schools and via media are important to achieve the global end TB goal of early detection and prevention of TB.

**Supplementary Information:**

The online version contains supplementary material available at 10.1186/s12913-023-10359-0.

## Introduction

Tuberculosis (TB) is an important public health concern, and it is one of the preventable causes of death in children [1]. The burden of TB infection in children aged 0–14 years is estimated to be about 12% of all TB cases worldwide [2]. In 2022, an estimated 1.3 million children fell ill with TB and 183 000 died of TB globally [2]. The World Health Organization (WHO) recommends TPT for all HIV-infected children and HIV-uninfected children younger than 5 years who are household contacts of bacteriologically confirmed pulmonary TB cases (child contacts) [1–3]. At the first UN high-level meeting on TB in September 2018, member states committed to ensuring that TPT be provided to at least 30 million people, of which 4 million should be children under 5 years of age however, these targets were not reached for the five-year period 2018–2022 [1, 2]. Treatment of *Mycobacterium tuberculosis* (Mtb) infection can reduce the risk of progression to TB disease in children [4, 5]. The uptake of TPT is sub-optimal globally [3, 6–8]. Despite the WHO recommendation to increase access to TPT in children under 5 years, its uptake is still low with a large proportion of children not initiated on treatment or not completing it [3, 4, 9]. In 2018, 1.3 million children under 5 years were estimated to be eligible for TPT however, only 27% of these children were reported to have been initiated on TPT [1]. Of the children reported to have been initiated on TPT, 266 040 (76%) were in Africa [1]. Though approximately 10% (25 357) of the children initiated on TPT in Africa were from South Africa, this represents about 59% of the children eligible for TPT in the country [1].

In South Africa, there is poor implementation of TPT guidelines and children are being lost along the TPT cascade of care [6, 7]. To date most studies on the uptake of TPT in children in South Africa have been conducted in the Western Cape. An evaluation to determine the proportion of child contacts under the age of 5 years who were initiated on and completed TPT was conducted in Cape Town, South Africa in 2011 across 14 primary health care (PHC) facilities [6]. This evaluation found that less than half (46%) of the eligible child contacts were screened for TB, 58% of the child contacts who were screened for TB were initiated on TPT and less than 14% of those initiated on TPT completed treatment [6]. Other studies on the management of child TB contacts in Sub Saharan Africa have also reported low proportions of eligible child contacts being initiated on TPT [4, 10–12]. The delivery of TPT may be overestimated because the number of contacts identified do not represent all eligible child contacts due to incomplete records thus the true denominator of contacts may be significantly higher [6]. It is well known that TPT uptake in children is not reaching the desired levels however there is a limited understanding on the barriers to increasing the delivery of TB preventive services to child contacts.

Barriers to increasing delivery of TB preventive services to child contacts exist at both the health system and patient level [3, 10]. Structural health system challenges, lack of access to TB preventive services, and lack of knowledge among both caregivers and healthcare workers contribute to the poor implementation and adherence to TB preventive strategies [3, 9, 10, 13, 14]. Countries with a high TB burden like South Africa need to prioritise scaling up access to TB preventive services in people centred care environments allowing for patients and caregivers to be involved in decision making [3]. Developments have been made to shorten TPT regimens and these include: child friendly formulations of rifampicin and isoniazid taken daily for 3 months (3RH); rifapentine and isoniazid taken weekly for 3 months (3HP) or daily for one month (1HP); and rifampicin taken daily for 4 months (4R) [15]. With the development of these new regimens for TPT there is a need to further understand the gap in delivery of TPT among child contacts and explore the barriers to increasing the delivery of TB preventive therapy to child contacts. Using a qualitative approach, we aimed to assess the barriers and motivators of initiating IPT in child contacts from the provider and caregiver perspective.

## Methods

### Study design and study setting

This qualitative study was conducted at two PHC clinics in the Ekurhuleni district, Gauteng, South Africa. The two clinics were purposively selected based on the following criteria: having TB case notifications greater than 100 cases per year; provided immunization, TB and HIV care baby wellness and family planning services; and had implemented the TB module within Tier.net. Tier.net is an electronic register of TB data integrated with HIV programme data. All health care services provided at each PHC were managed by a facility manager.

### Study population and participant recruitment

Facility managers at both PHC clinics were approached and invited to take part in the study and semi-structured questionnaires were administered to them. Each facility only had one facility manager. The facility manager had to have worked at the facility for at least 12 months to be eligible to take part in the study. Facility staff working in TB services (TB staff) at each facility assisted the facility managers in responding to the questionnaires.

Caregivers who were parents or legal guardians of children under five years who were at risk for TB accessing services at “high yield” PHC facility points such as: immunization, TB treatment, ART, baby wellness and family planning departments were randomly approached and invited to take part in face-to-face interviews.

### Ethics approval and consent to participate

The study obtained ethical approval from the University of Witwatersrand ethics board (Approval number: 180301) and the Ekurhuleni Health District Research committee. Facility staff, and caregivers were required to provide written informed consent before being enrolled into the study. The responses from the semi-structured questionnaires with facility staff were recorded in writing but the sessions not audio recorded. In-depth interviews with caregivers were audio-recorded and caregivers were required to provide written informed consent for the interviews to be audio-recorded. Participants were excluded from taking part in the interviews if they were unable to communicate in one of the study approved languages.

### Data collection

Data was collected from April to July 2019 by trained research assistants (BM, KS, NF and KM) and the co-investigator (RM). BM and KS were male whilst the rest of the data collection team was female. At the time of data collection, the entire team were employed by the Aurum Institute. Semi-structured interviews using a questionnaire (supplement 1) with facility staff were conducted by the co-investigator (RM). The facility managers answered a few basic questions and TB staff answered the TB specific questions. From the semi-structured questionnaire, we obtained informatin on the availability of TPT, human resources, clinic procedures, challenges, and experiences of delivering TPT and TB preventive services. A facility checklist (supplement 2) was used to conduct the facility observations. The semi-structured questionnaires were administered at the clinic in the health facility manager’s office. The facility checklist was also completed at the clinic and to record field notes.

Caregivers who accessed services at “high yield” PHC facility points were interviewed using a semi-structured interview guide developed following a review of literature (supplement 3). The interview guide was divided into themes aimed at understanding the enablers and barriers of accessing TPT services as shown in Table 1. The interview guide was piloted amongst a few participants and questions were revised before being finalised and used for data collection.

**Table 1:** Themes explored in in-depth interviews with caregivers

Perception of personal susceptibility to TB
Perception or experience of TB preventive services at public health facilities for children under five
Barriers to accessing TB preventive services for children under five
Enablers to accessing TB preventive services for children under five
Recommendations to improve accessing TB preventive services for children under five.

Caregivers were approached whilst they were at the clinic and appointments were set up for the interviews to be done at a convenient time for them. Approximate duration of the in-depth interviews was about 30 min. The interviews were conducted in a private and quiet space at the primary health care facility. Interviews were conducted in either English, isiZulu, Sepedi, Sesotho or isiXhosa and they were digitally recorded. During the interview, only the participant and the interviewer were present.

**Fig. 1:**
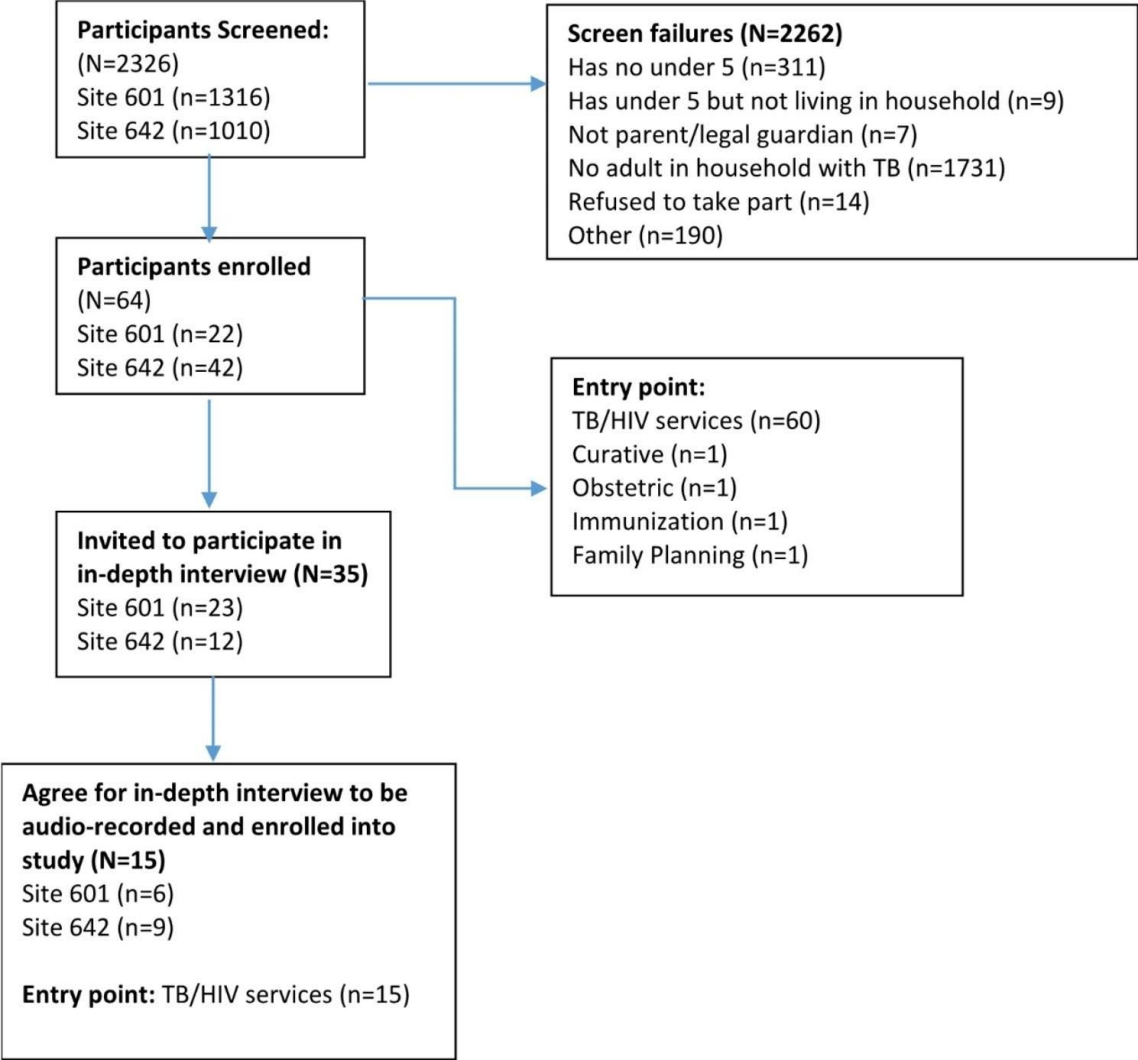
Summary of the participants approached and agreed to take part in the in-depth interviews

Two facility managers, one from each of the selected clinic took part in the study. TB staff at each clinic who were involved in TB care assisted the facility managers to complete the questionnaires. Figure 1 shows a summary of the caregivers approached and enrolled. Caregivers were defined as the parents of legal guardians of children under the age of 5 years. Of the 64 caregivers enrolled into the larger study which sought to assess the number of children under five in households at risk for TB and initiated onto IPT, fifteen caregivers aged between 18 and 43 years agreed to take part in the in-depth interviews. Thirteen (87%) of the participants were female. All the interview participants had one adult diagnosed with TB living in the same household and at least one child under the age of 5 years. The number of children under the age of 5 years per household ranged from 1 to 3 children. All the participants interviewed were accessing either TB treatment or HIV treatment services.

## Data analysis

The semi-structured questionnaires from managers and health workers were reviewed and the findings from both clinics were summarised. We looked for patterns and findings that either stood out for each clinic or were common. The recordings from the in-depth interviews with caregivers were transcribed verbatim and translated into English where applicable. A quality check was done by co-authors (FM and RM) on all the transcripts before the coding and analysis took place. QSR NVIVO 10 software was used to manage and analyse the data. Thematic analysis using a deductive approach was used to develop themes. The codebook template was developed by three members of the research team with a master’s and doctorate level qualification (FM, RM and CM).

To develop the initial codebook, RM and CM independently coded two transcripts each. The initial codes were based on the themes explored in the in-depth interviews as shown in Table 1. This was followed by cross-checking the codes, merging the broad coding into a finalized codebook through an iterative process. During this process, codes which were not common across the transcripts were cross-checked and merged if the definitions were similar. For example, the following codes were merged: “need to understand why prevention is better than cure”, “lack of information on TB screening and being aware on the existence of preventive therapy”, “TB Treatment and IPT”, and “location where information about TB screening for children given”. We merged these codes as all referred to a general lack of knowledge expressed by the caregivers about TB screening, prevention, and treatment for children. The merged code was renamed “lack of knowledge on TB transmission, screening, prevention and treatment for children”.

A third researcher with a master’s level qualification (FM) applied the final codebook to all the transcripts. Trends and patterns were discussed by the three researchers and some of the themes were refined. Following the process of refining and merging themes, all the 15 interviews started to reveal similar information therefore saturation was reached. Once there was mutual agreement on the themes amongst the three researchers, the themes were finalized. Direct quotes are presented in the results to express the key dimensions of each theme.

We used the COnsolidated criteria for REporting Qualitative research (COREQ) checklist for writing this manuscript (Supplement 4).

## Results

### Motivators and barriers to accessing IPT services from the facility staff perspective

Facility staff at both clinics felt that even though caregivers knew of family members who were on TB treatment, caregivers delayed bringing their children for TB screening and TPT. Where caregivers were not aware of family members on TB treatment, facility staff reported that it was due to the challenge of patients identified as index cases not disclosing their status to their household contacts or not informing clinic staff who their household contacts were. Where household contacts had been disclosed, facility staff faced challenges in tracing due to incorrect addresses recorded in patient files. Patients gave incorrect addresses and contact numbers because they were out of the clinic’s demarcation area and did not want it to be known as they would be asked to go to their local facilities.

The facility staff raised the issue of TB-related stigma as a challenge which may have discouraged index cases from disclosing their status. From the facility observations, it was evident that there was a lack of space at the clinic to allow for triaging of TB patients. The mixing of TB patients with other patients in the waiting areas was another challenge raised by facility staff, as caregivers may not have been comfortable with having their children remain in the waiting room with other TB patients. The facility observations also confirmed that there were TB screening tool shortages and IPT stock outs which negatively affected the provision TB preventive services to children at both facilities which was also reported by the facility staff.

According to the facility staff, some parents did not understand why their TB negative child needed to be started on IPT because the concept of prevention did not make sense to them. Parents would bring their children in for TB screening and for treatment of other conditions, but they would refuse IPT. The facility staff recommended creating awareness on TB preventive services through television adverts and sending SMS’s.

### Motivators and barriers to accessing IPT services from the caregiver perspective

Three themes emerged from the data. The dominant theme that emerged was that due to their caring nature, caregivers had the desire for their children to be protected from TB. The role of caregivers as a protective figure gave them the desire to access TB preventive services for their children. The second theme that emerged was that caregivers had the desire to be educated and caregivers revealed their preferences for education on TB preventive services and TB screening. The last theme that emerged was that caregivers had a lack of knowledge on TB, TB transmission and screening. This became a barrier and prevented them from accessing TB preventive services.

#### The role of caregivers as a protective figure

Caregivers expressed a desire for their children not to be infected with TB. Caregivers did not wish for their children to experience pain or physical distress due to the symptoms of the disease or die from it.

“They must be protected because TB kills and it’s very painful for child to be killed by TB and not even know how they got infected.” – ID2.

“I would love for them to get it (IPT) because it’s really painful to have TB” - ID3.

“Children must be protected and not infected.” - ID5.

Caregivers expressed their willingness to have their children screened for TB. They were committed to taking their children to the clinic for follow up visits after screening and to collect IPT.

“I also want them to be checked. If we find that they, have it, I must take care of them and help them take treatment from that time until they finish.”- ID4.

“It’s easy to come and collect my children treatment because I don’t want my children to be sick and not to get TB.” - ID1.

Caregivers who had taken their children for TB screening were relieved when they found out that their children had tested negative for the disease.

“I was happy because of I didn’t want them to go through what I went through because it was very painful.” – ID10.

“Yea that’s something that would make me happy. I mean I think I would feel ok because at the end of the day I know that this will reduce the risk of the child having TB. This would be a way for me to protect her from this disease. Yea I would feel ok with this thing.” – ID13.

#### Caregiver preferences for education on TB preventive services

Caregivers indicated that they wanted to learn about the importance of accessing TB preventive services. Most caregivers suggested that health workers should share more information on TB with them.

“Speaking of TB in children, I don’t want to lie but I don’t know that much but I think it’s about time they taught us. Yah even at clinics there should be that chance. When you go check for TB, they should use just five minutes to tell you about how dangerous TB is in children. And also, how they test them and why they need to test them that way. Cause of other people, they are going to get scared that you’re going to put a pipe in my baby’s stomach without explaining to what the importance of doing that is. Why should we have to do that? Is there another way? Or you should choose between this one and that one.” - ID8.

“So, you see knowledge (on TB, TB preventive services, and TPT ) is what we need most” – ID13.

Most caregivers felt that if they were educated on how children can be infected and affected by TB, they would be motivated and encouraged to bring in their children for TB preventive services.

“I think actually there should be a support group that will call parents and give them more information about TB so that they know what TB is, how it affects and spreads so that if they have information, they can have motivation to go and get their children tested for TB statuses”- ID8.

“I think you must teach parents about screening and about this pill that protects children also do door to door campaigns teaching community about TB.” – ID1.

Most caregivers indicated that they preferred for TB screening to be conducted at crèches and schools with some opting for it to be done in the household. Caregivers felt that crèches and schools were a good place as there were many children there and being screened at the household eliminated the need to travel to the health facility. Caregivers were not comfortable with traveling to the facility because of the distance and the fact that their children had to sit in the same waiting room as other TB patients. Caregivers also felt that crèches and schools were ideal for the provision of information on TB and TB preventive services. In addition to crèches and schools, caregivers suggested that information on TB and TB preventive services could be provided at clinics, in the media and in the community by running campaigns frequently.

“I also think that it’s important for the people checking to go to schools because that’s where most children are, in schools and crèches… If parents agree, then they check the children in the schools because in the townships, parents won’t just go to the clinic, maybe because of lines but it’s easy to get children tested in schools”- ID4.

*“To make things easy there must be nurses that will visit us and check children for TB at our homes. There is a distance from home to the clinic.” - ID1.*

“Maybe TB testing in families… In the clinics, they must make- I don’t know how they can do it; a ward, or a place around the clinic where the children would be checked cause here, we must bring them where old people who have TB are also sitting, which means that they are not safe.” – ID5.

“And they can launch campaigns for it cause people mostly, they can understand things through campaigns. There should be campaigns that speak about TB. Adults think that TB only infects adults and all of us, most of us, we don’t think that a child can get TB… There can be some small campaigns or at least they can speak about it at clinic and they… they encourage the testing of the kids. - ID10.

“Uhm, nurses must teach parents a lot about TB and then there must be pamphlets and billboards. There must also be people who will go crèches- Nurses must go around and teach mothers and staff.”- ID2.

#### Lack of knowledge on TB transmission, screening, prevention, and treatment for children

The most important barrier to accessing TPT was that the caregivers lacked knowledge on TB transmission, screening, prevention, and treatment in children. Caregivers did not understand how children could be infected with TB, where some thought only adults could get TB.

“I thought only old people can get TB because I didn’t have information about TB.” – ID8.

“I would not know; I was also surprised how she was infected, or she got it from her father because he was coughing.” – ID6.

“We have the belief that the only important thing to check on children is HIV/AIDS not TB. And they (caregivers) have this belief that where would they be getting TB from? They don’t use (chemicals), they don’t use these things they don’t use those things. It’s because we don’t know what causes one to get infected with TB. Even if they don’t work with chemicals, even if they don’t work with some things, and they don’t smoke cigarette. Mostly, we like saying that people get TB because they smoke cigarettes. They will say no, the child does not smoke cigarettes, how can they get TB if they don’t smoke cigarettes”- ID10.

Some caregivers did not know how children were screened for TB and others did not understand the value of TB screening and preventive therapy in children. The complexity process of screening was not fully understood by caregivers.

“No, I had never heard of that, that there is a way that they screen children for TB.” – ID13

“No! For children they must not be given pills every day because it would be an overdose and their system will be full of this thing, they can maybe get an injection.”- ID11.

“No. I don’t have information at all. Because of they had also told me at the clinic that I also have to bring mine so that they can put in some pipe inside the stomach before he eats anything, with no milk, anything, on an empty stomach. That’s what I know. And it scared me. I thought it’s very risky to put a pipe inside like inside the mouth, that goes into the baby’s intestinal tract. I don’t know but I have never seen them do it but it’s what they explained to me. I just thought it’s, I thought it’s very risky. Maybe they can even vomit. I know that they said they must not eat at all.”- ID9.

## Discussion

In this qualitative study we describe the barriers and motivators of finding and screening child contacts under 5 years from the provider and caregiver perspectives. Our study provides evidence that caregivers delayed taking their children to the clinic even though they had the desire for their children to be screened for TB. Both caregivers and facility staff felt that the lack of knowledge on TB transmission, screening, prevention, and treatment for children was a major barrier for caregivers preventing them from accessing TB preventive services. However, caregivers had the desire to be educated on TB preventive services and facility staff felt that educating caregivers could have a positive impact on improving access to TB preventive services.

The lack of knowledge as a barrier to accessing TB preventive services is consistent with findings in other studies on the management of child contacts of TB patients [16–19]. Other studies in sub-Saharan Africa have recommended that caregivers be educated on the importance of TB preventive services [7, 13] which is consistent with our findings from both the semi-structured questionnaires with facility staff and in-depth interviews with the caregivers. Both caregivers and facility staff recommended educating the community on issues related to TB which implies that the current methods used to educate people on TB are not effective. Educating the community on TB and TB preventive services is important because it can empower and encourage the community to become actively involved in the fight against TB as it provides them with information. The need for more people-centred approaches in healthcare programmes is widely recognised therefore educating caregivers is an important tool that can be used to encourage access to TB preventive services [20] thus increasing the uptake of TPT.

In South Africa, health education including education on TB is done primarily by health workers and it is included in the services provided at PHCs [20]. Patients also receive some education in the media and through the distribution of health materials which are also displayed at PHCs [20]. The Road-to-Health booklet (RtHB) is a standardised national tool for growth monitoring and the assessment of health among children from birth to five years of age and is used by ward based outreach teams (WBOTS) [21, 22]. WBOTS are made up of community health workers (CHWs) and led by a professional nurse [21, 22]. The RtHB includes monitoring and providing health education on preventive and treatment interventions for HIV and TB amongst other infectious diseases affecting children [22]. The findings of our study suggest that there may be gaps in the methods of providing education or that caregivers are unable to access and understand information on TB preventive services. An assessment on the utilisation of the RtHB for the monitoring on child nutrition in the Western Cape, South Africa found that CHWs encountered barriers in the implementation of the booklet as a monitoring and education provision tool [22]. CHWs lacked training, encountered language barriers when communicating with caregivers and had insufficient time to complete all tasks [22]. Though this study was specific to the monitoring of child nutrition, CHWs may face similar challenges in the use of the RtHB as a tool to educate caregivers on TB preventive services and TPT. These gaps in the provision of information may explain why caregivers and facility staff suggested that there is a need for more education on TB preventive services for both children and adults despite what is already being done.

Caregivers suggested additional ways of spreading information on TB such as educating parents at crèches and schools and conducting campaigns in the community. These ways in addition to health education provided at the PHC level will be useful to ensure that information reaches the community and people who may not visit clinics. Educating the community on TB may also have a positive effect on the issues raised around TB related stigma by the facility staff as a barrier to accessing TB preventive services. Index cases were afraid of disclosing their status to their household members due to stigma and as a result, caregivers may not have been aware of the need to take the children in the household for TB screening. There is a need for TB programmes to consider interventions that will help with the reduction of TB related stigma. Educating the community on TB may also influence index patients to give correct addresses enabling efficient contact tracing which is a challenge identified by facility staff. Health facilities may also consider requesting official documents which can be issued by ward councillors as proof of residence when patients register for care at their facilities. TB programmes could consider using social media, radio, and television to spread information on TPT [23]. There is increasing evidence that social media, radio, and television can be powerful tools for health promotion [23]. Whilst these tools may improve the spread of information, the messages need to be tailored for different audiences to be effective [23].

It is also important to consider how health care providers communicate with patients/caregivers to educate them on TB as our findings show that even though caregivers are being given information at the clinic, this information may be misunderstood. A pilot study in Lesotho exploring the preferences of child contact caregivers found that sometimes when patients/caregivers ask questions, the responses from the health care provider may be incomplete [13]. Responses may be based on the health care provider’s judgement on the patient/caregiver’s capacity to comprehend the information they are being given and this may intimidate patients or caregivers [13]. This may explain why the facility staff in our study felt that the caregivers delayed bringing their children for screening, yet they were aware of index patients at their households. It is therefore important to ensure that when health workers provide information, it is simplified and that the recipients have understood the information.

Caregivers have a protective role over their children which was expressed by their desire for their children not to experience pain due to TB infection, the commitment to ensuring that their children receive and complete TPT, and their recommendations for children to be screened even at crèches and schools and in the household. It is acknowledged that caregivers have an important role to play in affecting the uptake of interventions for children. Even though caregivers may express the desire to be involved and empowered in making decisions for their children to access treatment, they often did not do so implying that other barriers existed. These findings are consistent with the findings from a pilot study which explored the preferences among caregivers of children in Lesotho [13]. To increase the uptake of TPT in children, it is important to understand how health care providers can work together with caregivers to make them understand its importance and influence their behaviour.

## Strengths and limitations

Few qualitative studies have been conducted to date exploring the barriers of accessing TB preventive services from the caregiver’s perspective. One of the strengths of our study is that it explored the perspectives of caregivers in-depth which provided rich data. The facility observations allowed us to understand the context in which the facility staff were being interviewed. For TB preventive programmess to be successful, both the provider and patient aspects need to be considered. Our study explored both the health system and patient perspectives allowing us to give insight into how future TB preventive programmess may be designed.

Our findings are limited because the study was conducted only at two health facilities in one district. Thus, we recommend that the attitudes and perspectives of other health workers from other districts and facilities be also explored. The study collected data related to human behaviour making it prone to bias such as social desirability as participants try to offer response that are socially acceptable. To mitigate the risk of bias, participants were assured that their responses would be kept confidential, and the indirect benefits of the study were emphasized.

The study was conducted in 2019, before the shorter TPT regimens were available therefore our findings may have reduced relevance to the current practice as these regimens are shorter and have better completion rates than IPT. Despite this, our study still provides valuable insights on aspects of TPT delivery that must be considered to improve uptake regardless of the regimen.

## Conclusion and recommendations

The delivery of TPT in children under the age of 5 years is limited by system level factors including stock outs, shortage of screening tools, and lack of space at health facilities. Individual level barriers such as TB related stigma, and lack of knowledge on TB preventive services including TB transmission in children also results in a reduced uptake of TPT delivery. Health education on TB transmission, screening, and TPT should be conducted at a community level, clinics, crèches, schools and via media in addition to the PHC level. Health education could also be targeted at index cases when they are started on treatment. The importance of disclosing one’s status to household contacts should be re-enforced to index patients when they visit health facilities to collect their treatment. TB prevention programmes should raise awareness of TB preventive services and consider addressing both system level factors and individual barriers affecting the uptake of TPT in child contacts.

### Electronic supplementary material

Below is the link to the electronic supplementary material.

Supplementary Material 1: Semi-structured questionnaire for facility staff

Supplementary Material 2: Facility checklist

Supplementary Material 3: Semi-structured in-depth interview guide for caregivers

Supplementary Material 4: COREQ checklist

## Data Availability

Data and materials are available and requests for these can be sent to the corresponding author.

## List of abbreviations

AIDS: Acquired Immune Deficiency Syndrome
ART: Antiretroviral treatment
CHW: Community Health Worker
HIV: human immunodeficiency virus
HIV: Human Immuno–deficiency Virus
IPT: Isoniazid Preventive Therapy
Mtb: *Mycobacterium tuberculosis*
PEPFAR: President’s Emergency Plan for AIDS Relief
PHC: Primary Health Care
RtHB: Road–To–Health booklet
SMS: Short Message Service
TB: Tuberculosis
TPT: Tuberculosis Preventive Therapy
UN: United Nations
WBOTS: Ward Based Outreach Teams
WHO: World Health Organization
