# Supplementary material for: “It’s about time they taught us”: a qualitative study evaluating the barriers to finding and screening child contacts under five at risk for TB in Gauteng Province, South Africa from the provider and caregiver perspectives
Source: BMC Health Serv Res. 2023 Dec 15;23:1415. doi: 10.1186/s12913-023-10359-0 (PMC10722712; doi:10.1186/s12913-023-10359-0)
Supplement: Supplementary file 1 — Supplementary Material 1: Semi-structured questionnaire for facility staff [file 12913_2023_10359_MOESM1_ESM.pdf]

A cross sectional study investigating the feasibility of identifying children under five at risk of TB in Gauteng Province, South Africa

SL02 FACILITY MANAGER QUESTIONNAIRE

SECTION 1: TO BE FILLED IN BEFORE VISIT

**1. Site number**	
**2. Date of assessment**	
**3. Person conducting the assessment**	
**4. Initial and Job title of person responding to questions**	

5. From the routine data complete the table for the last 12 months, children seen in this clinic *(indicate which months)*

	Mth/yr	Total number children under five seen	Total number children under five screened for TB	Total number children under five eligible for IPT	Total number children under five initiated on IPT	Total number children under five completed IPT	Total number children under five diagnosed with TB
	A	B	C	D	E	F	G
1							
2							
3							
4							
5							
6							
7							
8							
9							
10							
11							
12							

If no children – tick here ☐

Availability of staffing

6. Indicate numbers <u>Full time staff</u> of each category providing <u>TB services to children under five</u> in this facility (fill in only the appropriate line) . Please can you indicate number of FTEs per area.

Category	A. No of staff	B. No of staff that see adult patients?	C. No of staff that see child patients?	D. FTE<br>1 day/week = 0.2<br>2 days/week = 0.4<br>Half days 5xwk = 0.5	E. Full Time or Part Time
1. Site manager (if not doctor or nurse					
2. Doctors					
3. Professional nurses					
4. Enrolled nurses					
5. Enrolled nursing assistants					
6. Lay counsellors					
7. Pharmacist					
8. Pharmacy assistant					
9. Dietician					
10. Social worker					
11. Psychologist					
12. Clerical staff/data capturers					
13. Other: specify……………					

Staff turnover and unfilled posts

7. No of vacancies (budgeted but not in place) currently in this facility? ………..…………..……… ☐ ☐

8. How many staff members have left this facility in the past year? ………..…………………...…. ☐ ☐

9. How many new staff members in the past year? …………..…………………………………..…. ☐ ☐

10. Indicate numbers of people left and came to this facility in the past year?		
**Category**	**A. No of staff left**	**B. No of staff joined**
1. Site manager		
2. Doctors		
3. Professional nurses		
4. Enrolled nurses		
5. Enrolled nursing assistants		
6. Lay counsellors		
7. Pharmacist		
8. Pharmacy assistant		
9. Dietician		
10. Social worker		
11. Psychologist		
12. Clerical staff/data capturers		
13. Others: specify………		

Availability of essential services

11. Is the facility able to provide the following services? (fill in every line)		
**Service**	**A. Available Yes=1 No=0**	**B. No of days per week available**
1. HCT		
2. PMTCT		
3. HIV wellness service		
4. ART initiation		
5. ART follow-up/maintenance		
6. Curative care (minor ailments)		
7. Antenatal care		
8. Obstetric deliveries		
9. TB treatment		
10. Chronic disease care		
11. Post sexual assault services		
12. Home based care		
13. Support for obtaining grants		
14. On-site pharmacy		
15. TB sputum microscopy		
16. TB sputum culture		

17. X-ray facilities		
18. Immunization		
19. Feeding support (eg. Breast-feeding)		
20. Antenatal classes		

12. Regarding your opening hours at this facility, indicate when open and when patients need to arrive to be seen by a doctor on the same day and have bloods taken on that day.

	A. From [00:00]	B. To [00:00]	C. Bloods on the same day (Time 00:00)	D. Seen by doctor on the same day (Time 00:00)
1. Monday				
2. Tuesday				
3. Wednesday				
4. Thursday				
5. Friday				
6. Saturday				
7. Sunday				
8. Public Holidays				

13. Do you see patients who arrive without an appointment? 1=Yes, 0=No ☐

14. If yes, please list the services for which you see patients without an appointment

……………………………………………………………………………………………………….

………………………………………………………………………………………………………..

……………………………………………………………………………………………………….

Other information

………………………………………………………………………………………

………………………………………………………………………………………

………………………………………………………………………………………

………………………………………………………………………………………

………………………………………………………………………………………

………………………………………………………………………………………

SECTION 2: INTERVIEW

Pathways of care
(ask the clinic manager to relate to you what happens in each of these circumstances)

15. What normally happens when a person attending this clinic is suspected of having TB or requests a TB test?		
Where do they receive TB screening?		
If you suspect TB what do you do and where do they go?		
What TB investigations take place?		
If confirmed of TB, what happens next?		
What contact tracing procedures (if any) are followed in the facility?		
Where do your TB patients most commonly come from? (e.g. referred after admission, from general OPD, ANC,HCT)		
Pathways of care elaborated/available in writing	Yes	No

Referral and integration

16. If you don't offer this service, how would you describe your relationship with the following services?

Service	Good=1 Fair =2 Poor=3 4=NA	Detail
a) HIV testing		
b) TB Treatment		
c) PMTCT/antenatal Care		
d) Referral hospital		
e) Obstetrics		

17. Have you ever referred a patient to, or had contact with, the following providers?		
**Provider**	**Referred only=1<br>Contacted only =2<br>Referred and contacted =3 Never =0**	**Details**
a) A traditional healer		
b) Another private clinic or private GP		
c) A community organisation		
d) Social workers/ Department of Social Development		
e) Schools/education dept		
f) Birthing Assistant/Mid-wife		

Adherence management

18. I'd now like to ask you how you manage treatment preparation, adherence and tracking of IPT patients who don't attend follow-up appointments

Step/procedure	Description
Describe the steps (verbal or written) or procedures involved in preparing children under five for IPT	
Describe how you ensure that children under five remain on (adhere to) IPT?	
How do you monitor IPT in individual children under five?	
How do you track defaulters from the service?	

19. Appointments are made for patients attending follow-up TB services

	Yes=1 No=0
1. For return dates	
2. For return dates and times	
3. Patients can request appointment changes if dates don't suit them.	

20. Have there been any changes in the site in the past 3 years:

	Yes=1 No=0	Details
1. Management		
2. Staff members		
3. Facilities		
4. Services provided		
5. Types of patients		

21. What are the biggest challenges facing you as manager of this facility?

……

……

……

……

……

……

22. What are the biggest challenges facing your staff in this facility?

……

……

……

……

……

……

23. What are the challenges in delivering TB services in this facility?

……

……

……

……

……

……

……

24. What are your experiences of delivering TB services to children under five in this facility?

……

……

……

……

……

……

COMPLETED	VERIFIED	Date	Any comments
|___|__	|___|__	|___|___|/|___|___|___|/20|___|___|	
