# Supplementary material for: “It’s about time they taught us”: a qualitative study evaluating the barriers to finding and screening child contacts under five at risk for TB in Gauteng Province, South Africa from the provider and caregiver perspectives
Source: BMC Health Serv Res. 2023 Dec 15;23:1415. doi: 10.1186/s12913-023-10359-0 (PMC10722712; doi:10.1186/s12913-023-10359-0)
Supplement: Supplementary file 2 — Supplementary Material 2: Facility checklist [file 12913_2023_10359_MOESM2_ESM.pdf]

A cross sectional study investigating the feasibility of identifying children under five at risk of TB in Gauteng Province, South Africa

SL03 - FACILITY ASSESSMENT (to be filled in by research staff)

Source: PERFORMANCE STANDARDS FOR ANTIRETROVIRAL THERAPY, DECEMBER 2006, developed by JHPIEGO/Johns Hopkins University in partnership with the National Department of Health of the Republic of South Africa and the Foundation for Professional Development

01. Date of data collection.. ☐☐/☐☐☐/☐☐☐☐

DD / MMM / YYYY

02. Study Site number .... ☐☐

03. Type of health facility .... ☐

1… General practitioner (sole practitioner)
2… Group practice
3….NGO clinic
4… Public sector clinic
8…Other (please specify): ______________________

04. Location of facility.. ☐

1… City
2… Town
3… Informal settlement area
4… Rural / farming area
8… Other (specify) ______________________

05. Infrastructure for services 0=No 1=Yes

01…Dedicated counseling rooms.. ☐

02…Dedicated HCT testing rooms .... ☐

03…Dedicated observations areas (vital signs) .... ☐

04…Wellness / ART consulting rooms.. ☐

05…Blood drawing areas.. ☐

06…Pharmacy.. ☐

07…Dispensing room (if no Pharmacy) .... ☐

08…Staff tea room/kitchen.. ☐

09…Administration area/office ……………………………………………………………………………… ☐

06. Waiting area and reception for patients 0=No 1=Yes

1. Is there a waiting area for patients……………………………………………………………… ☐

   If there is a waiting area:

2. There is an adequate number of chairs for the number of patients present………………………… ☐
3. The temperature of the room is comfortable ……………………………………………………… ☐
4. Is there a dedicated waiting area for coughing patients……………………………………………… ☐
5. The floors are clean……………………………………………………………………………… ☐
6. Is the area well ventilated ………………………………………………………………………… ☐
7. The room has adequate lighting…………………………………………………………………… ☐
8. There is sufficient space for patients and staff to move freely ………………………………… ☐
9. There is a telephone for the reception clerk………………………………………………………… ☐
10. There is drinking water available ………………………………………………………………… ☐
11. Is there HIV/TB brochures/pamphlets available for patients………………………………………… ☐
12. Are there posters with HIV/TB messages ………………………………………………………… ☐
13. Masks available for coughing patients……………………………………………………………… ☐

07. Observations room 0=No 1=Yes

1. There is a desk and chair for each nurse/counselor ……………………………………………… ☐
2. There are chairs for patients for each nurse/counselor……………………………………………… ☐
3. There is a functioning scale………………………………………………………………………… ☐
4. There is a height board for adults ………………………………………………………………… ☐
5. There is a height board for children………………………………………………………………… ☐

6. There is a functioning blood pressure cuff and sphygmomanometer or blood pressure machine (in this area or in the consultation room) ……………………………………………………………… ☐
7. There is a functioning thermometer ……………………………………………………………… ☐
8. There is urine dipstix available ……………………………………………………………… ☐

08. Consultation rooms 0=No 1=Yes

1. There is adequate supply of equipment (examination lamp, functioning ENT set, opthalmoscope, reflex hammer and x-ray viewer, all present)……………………………………………………………… ☐
2. There are adequate supplies (gloves, soap, tongue depressors, cotton wool, all present)……………… ☐
3. There is adequate furniture (examination couch, desk and chair)………………………………… ☐
4. There are working hand washing facilities (in room) ……………………………………………… ☐
5. There is a sharps container in all consultation rooms …………………………………………… ☐
6. Outdoor sputum collection area and/or dedicated sputum collection area……………………… ☐

09. Counselling rooms 0=No 1=Yes

1. There is sufficient furniture (desk and chairs for counsellors and patients) ……………………… ☐
2. There are information and education materials …………………………………………………… ☐
3. There is storage available for patient notes/materials…………………………………………… ☐

10. Toilet facilities used by patients 0=No 1=Yes
(examine all the patient toilets and answer if present for all toilets)

1. Are there toilet facilities for patients……………………………………………………………… ☐
   If there are toilet facilities for patients:
2. Can the door lock from the inside…………………………………………………………………… ☐
3. Are the basins and taps in working order………………………………………………………… ☐
4. Is there soap in the toilets……………………………………………………………………………… ☐
5. Are there paper towels ………………………………………………………………………………… ☐

6. Is there a dustbin in the toilets…………………………………………………………………………☐
7. Does the toilet flush ………………………,,,,………………………………………………………☐
8. Is there toilet paper in the toilets……………………………………………………………………☐
9. The toilets are clean…………………………………………………………………………………☐
10. Are there dedicated toilet facilities for staff……………………………………………………☐

11. HIV Counselling and testing (HCT) and ART 0=No 1=Yes

1. Is HCT testing done in this facility…………………………………………………………………☐
   If HCT is being done:
2. Is HCT done in dedicated rooms (as opposed to general consulting rooms) ……………………☐
3. Does TB screening take place in the HCT rooms…………………………………………………☐
4. Is IPT dispensed in the ART rooms…………………………………………………………………☐

12. Record keeping 0=No 1=Yes

1. All clinic patients are appropriately identified in the files……………………………………☐
   (Verify in three random patient records that the following information is documented: patient number, ID number and patient name and/or number is on each page in the file)
2. There is a system to file patient records:
   a. There is a well-defined system for filing medical records systems………………………☐
   b. There is a well-defined system of collecting files from consultation rooms for temporary storage before filing…………………………………………………………………………☐
   c. Filing is up-to-date within 2 days of patient attendance……………………………………☐
3. The system ensures confidentiality of patient files and information
   a. Written policy and guidelines referencing confidentiality of medical records and patient information exist…………………………………………………………………………………☐
   b. All staff including volunteers sign a confidentiality code of conduct pertaining to confidentiality of medical records and patient information systems………………………………☐
   c. Hard copy files are locked up ……………………………………………………………………☐

d. Electronic patient files are accessed via password………………………………………………………☐

e. There is controlled access to the fax machine/receiving area of patient results…………………☐

3. Standardised forms are available for any new/repeat consultations…………………………………………☐

4. There is a system for documenting referrals ………………………………………………………………………☐
(Referrals, "transfer in" and "transfer out" and referral letters are documented correctly (reconfirm by reviewing one transfer file, A copy of the referral letter is kept in the file)

5. Verify with the reception clerk and the social worker that: there is a documented protocol to manage patients that miss appointments (defaulters) ……………………………………………………………………☐

6. Patients who miss a treatment appointment are documented in a register……….. ………………………☐

7. A dedicated person contacts the patient after 2 days of the missed appointment (telephone or home visit) ..☐

8. Site has a functioning fax machine or email ……………………………………………………………………☐

9. Site has a printer and copier……………………………………………………………………………………☐

13. Educational Materials

1. The site has educational materials available (0=No 1=Yes) ………………………………………☐

If there is are educational material, answer the following questions:

2. Are they up to date and relevant ……………………………………………………………………☐

3. Are they in the appropriate languages……………………………………………………………………☐

14. On site Data management

1. The site has an electronic register (0=No 1=Yes) ……………………………………………………☐

If there is an electronic register, answer the following questions:

4. Computer and software for data capture are available and functioning ………………..………...………..☐
5. The patient's electronic file is complete (Verify in the electronic data base for 3 patients, if no electronic file – NA=7)…………………………………………………………………………………………………..……☐
6. Verify that the data from the group of patients seen one week ago is entered in electronic file (if no electronic file NA=7)…………….…………………………………………………………………...………..............………☐
7. The data capturing room has adequate lighting and comfortable with regards temperature and ventilation…………………………………….……………………...………………..……………………..……☐
8. The data capturing room has a desk and chair for the data capturer……....……..…………………………☐

15. Dispensing room (0=No 1=Yes**)**

1. An appropriate place for storage of daily stocks and dispensing of drugs exists ……………………..…..…☐
   (Area is adequate for the amount of drugs and for dispensing of drugs to clients, privacy for dispensing of drugs is guaranteed, there is adequate ventilation, area is organized and clean, it is protected from sunlight and it is not humid)
2. Area is secure and access is controlled…………………………………………………………….……….…..…☐
3. Drugs stored on shelves are properly labeled (easy to find, not mixed up)...………………..…………..…☐

16. PROTOCOLS

1. Check the availability of the following in the TB and ART/HIV service

Protocol	A.Available Yes=1 No =0	B.National guideline Yes=1 No=0 NA=7
1. South African National TB Guidelines 2017 - Children		
2. National TB Management Guidelines		
3. TB Screening tool (most recent)		
4. Flow charts on TB diagnosis – New patients (blue) (either in TB control guidelines book or on a clinic/CHC wall, nurse should show it to you)		
5. National strategic plan for South Africa 2017-2022		
6. Guideline on TB infection control		
7. Guidelines on Immunization		
8. Prevention and treatment of opportunistic and HIV related diseases in adults (non-ART adult guidelines)		
9. Managing HIV in children		
10. Prevention of mother-to-child HIV transmission and management of HIV positive pregnant women		

COMPLETED	VERIFIED	Date	Any comments
|___|__	|___|__	|___|___|/|___|___|___|/20|___|___|	
