# Supplementary material for: “It’s about time they taught us”: a qualitative study evaluating the barriers to finding and screening child contacts under five at risk for TB in Gauteng Province, South Africa from the provider and caregiver perspectives
Source: BMC Health Serv Res. 2023 Dec 15;23:1415. doi: 10.1186/s12913-023-10359-0 (PMC10722712; doi:10.1186/s12913-023-10359-0)
Supplement: Supplementary file 3 — Supplementary Material 3: Semi-structured in-depth interview guide for caregivers [file 12913_2023_10359_MOESM3_ESM.pdf]

# A cross sectional study investigating the feasibility of identifying children under five at risk of TB in Gauteng Province, South Africa

## SL04 IN-DEPTH INTERVIEW GUIDE

## PART I: STRUCTURED QUESTIONNAIRE

### *Introduction*

Staff to say out loud before starting interview:

- Thank you for agreeing to take part in our research
- We are interested in knowing about reasons that influence parents/legal guardians to access TB services for their children. There are no right or wrong answers. <u>You do not have to answer any questions if you don't want to.</u> Just say pass and I will move to the next question.
- Your answers to these questions may help us improve TB prevention services.
- All of your answers will be kept confidential. Confidential means we will not tell your answers to anyone outside the research group.

**DATE OF INTERVIEW:**

**INTERVIEWER:**

***Time started (HHMM):***

***Questions***

1. What is your gender?
2. How old are you?
3. Where were you born?
4. What is your educational status?
5. Are you employed?
6. What is the name of the public health clinic that you attend?
7. How far is the clinic from your home?
8. How many children under five do you have?
9. Have you ever taken your children under five for TB screening?
10. Have you or anyone in your household screened for TB?
11. Are you or anyone in your household on TB treatment?

***Time ended (HHMM):***

## PART II: IN-DEPTH INTERVIEW

### *Introduction and Ground Rules*

1. Obtain written informed consent first, before any data are collected.

2. Interviewer to introduce themselves. Thank you for taking the time to meet with us today. Our names are [*insert names*] _____________ and we would like to talk to you about TB services for children. We are doing this project to understand how we might improve TB services for children under five, and particularly to see whether there are better ways to encourage parents/legal guardians to take children for TB screening. We are interested in your views regardless of your experience with TB services for children under five. We want you to be as open and honest when answering. There are no right or wrong answers in this discussion. Please feel free to tell us what you think.

3. Interviewer to explain the ground rules and terms of confidentiality for the interview:

    - The participant does not have to answer any question they do not want to.
    - The information you share will be handled in confidence.
    - When we report back on the information collected in this discussion, your comments will not be able to be linked to you specifically.
    - We ask that you also agree not to share anything discussed in this room with others.

4. The discussion should take 45 minutes to one hour.

5. Interviewer to inform the interviewee that the in-depth interview will be audio-recorded to make sure that all themes are captured. Turn the audio-recorder on and ask for verbal permission again to audio-record, while the audio-recorder is running to verbally capture consent (this is a double check against the written consent). We will be recording the session because we don't want to miss any of your comments. Although one of us may take some notes while we talk, we can't write fast enough to get everything down on paper. As we are recording, please try to speak loudly so that we don't miss your comments.

***Themes to be explored***

- Perception of personal susceptibility to TB
- Perception or experience of TB preventive services at public health facilities for children under five
- Barriers to accessing TB preventive services for children under five
- Enablers to accessing TB preventive services for children under five
- Recommendations to improve accessing TB preventive services for children under five.

***Time started (HHMM):***

***Questions***

1. How can a person become infected with TB?
2. What did you know about TB before you/the adult in your household tested positive?
3. I would like to hear about your experience accessing TB preventive services. When did you/the adult first test TB-positive? Where did you/the adult test positive? How did you feel when you were told the test results?
4. Tell me about the treatment. Did you/the adult find it difficult to take the medication as directed? Why or why not?

*Now I'd like to talk about children and TB.*

1. What do you think places a child at risk for TB?
2. What do you know about screening children for TB? Where did you learn this information? [If clinic not mentioned]: Has the clinic ever talked to you about screening you child[ren] for TB?
3. What are some reasons why you think children should be screened for TB?
4. Can you tell me about your understanding of screening children under five for TB? That is, how are children screened for TB? (Probe on prevention and treatment)
5. Can you explain to me your understanding of Isoniazid preventative therapy (IPT) for children? Where did you hear about it? In your own words, what does that mean? How long is the dose and how is it given to children?
6. How do you feel about your children having to take daily treatment for preventing TB for at least 6 months? What are your thoughts about how TB preventive therapy could be improved?

7. Have your child(ren) ever been screened for TB?
    - If Yes: I would like to hear your thoughts and experiences of taking your children to be screened for TB. Where were they screened and how? What were the results? How did you feel? How did you feel about the service you received at the clinic?
        i. In your opinion, what is positive about the current TB testing services for children under five?
        ii. In your opinion what are negative aspects of the current TB testing services for children under five?
    - If No: Why have you never tested your child(ren) for TB? How would you feel about taking your children to be screened for TB? Do you think there are any benefits to screening children for TB? What might make it easier for you to bring your child(ren) to the clinic for TB screening?
8. In your opinion, what types of methods could be used to inform parents/legal guardians of options for screening their children for TB?
9. Sometimes children who are exposed to TB do not get appropriately screened for the infection. Why do you think this is? What could make it easier for parents/legal guardians to have their children screened for TB?

***Any other comments***

Are there any final thoughts you have about TB or TB screening of children under five?

***End of session***

Now we have come to the end of our discussion. Thank you for your participation. If you have any questions about your study participation, please contact us. Thank you.

***Time ended (HHMM):***
